# Innate sensors for nucleic acids and lupus pathogenesis

**DOI:** 10.1186/ar3971

**Published:** 2012-09-27

**Authors:** AN Theofilopoulos, R Gonzalez-Quintial, Y-T Koh, R Baccala, DH Kono

**Affiliations:** 1 The Scripps Research Institute, La Jolla, CA, USA

## 

We continue our efforts to define the pathogenesis of systemic lupus-like autoimmunity in predisposed mouse models by focusing on the role of endosomal nucleic acid-sensing TLRs.

We reported that NZB mice deficient for the common receptor for type I IFNs showed significant reductions in all disease parameters. To differentiate whether the pathogenic effects were mediated by the multiple IFNα subtypes and/or the single IFNβ, we created congenic NZB mice lacking *Ifnb *and found that disease severity was unaltered, strongly implicating IFNα subtypes as the principal effectors. We then documented that long-term treatment of male BXSB mice with an anti-IFNAR antibody of mouse origin reduced serologic, cellular and histologic disease manifestations and extended survival, suggesting that disease acceleration by the *Tlr7 *gene duplication in this model is mediated by type I IFN signaling. The efficacy of this treatment was greater when applied relatively early in the disease process, but reductions in some disease characteristics, especially kidney pathology, were evident even when treatment was initiated at later stages, and a transient therapeutic effect was also noted in the MRL-*Fas^lpr ^*model. The combined findings suggest that antibody-mediated IFNAR blockade may be a useful treatment approach in human SLE and probably other autoimmune syndromes in which these cytokines appear to play a pathogenic role.

We have also hypothesized that engagement of nucleic acid-sensing TLRs may be responsible for spreading the aberrant response beyond ANAs to encompass the broader spectrum of disease-associated autoantibody specificities. Using MRL-*Fas^lpr ^*mice congenic for the *3d* mutation of the *Unc93b1 *gene, in which signaling by all endosomal TLRs (TLR3, TLR7, TLR9) is extinguished, we indeed found reductions not only in autoantibodies against nucleic acids and associated proteins (anti-chromatin, anti-RNP, anti-Sm), but also in a broad panel of autoantibody specificities, particularly against antigens known to contain or bind to nucleic acids, such as cardiolipin, β_2_-glycoprotein 1 and myeloperoxidase. Surprisingly, even anti-erythrocyte autoantibodies and hemolytic anemia were significantly reduced in NZB mice congenic for the *3d* mutation compared with wild-type controls. Thus, almost the entire gamut of autoantibodies in lupus can be traced to the initial engagement of nucleic acid-sensing TLRs.

To examine whether engagement of B-cell intrinsic nucleic acid-sensing TLRs is required for autoantibody production *in vivo*, we generated mixed bone marrow chimeras of B6-*Fas^lpr ^*mice that were either *3d*/IgH^b ^or WT/IgH^a ^and measured allotype-specific IgM RF and IgG_2a _anti-chromatin responses. Strikingly, both autoantibodies were derived almost exclusively from the wild-type donor B cells. This finding underscores the essential significance of B-cell nucleic acid sensors in loss of tolerance and production of autoantibodies in lupus and provides a specific target for intervention.

Evidence suggests that plasmacyotoid dendritic cells (pDC), the major producers of type I IFNs, are likely to be involved in the pathogenesis of lupus. One ENU phenovariant identified by Beutler and colleagues, named *feeble*, showed abrogation of both TLR7-induces and TLR9-induced type I IFN and proinflammatory cytokine production by pDCs, while leaving intact pDC development and TLR responses by other cells. The *feeble *phenotype was mapped to a mutation in *Slc15a4*, which encodes the peptide/histidine transporter 1 (PHT1), one of the four members of the solute carrier 15 (Slc15) family of proteins. In preliminary collaborative studies with Beutler and colleagues, lupus-prone B6*Fas^lpr ^*mice congenic for the *feeble *mutation showed significant reduction in hypergammaglobulinemia, lymphadenopathy and mortality. These findings provide direct evidence for the role of pDC in the pathogenesis of systemic autoimmunity and suggests that pharmacologic agents that interfere with *Sle15a4 *function may be useful in treating lupus and other diseases in which these cells appear to be involved.

